# *miR-145-5p* Is Required for the Antitumor Activity of *Strophanthus gratus*-Derived Ouabain in Colorectal and Breast Cancer

**DOI:** 10.3390/ph19071099

**Published:** 2026-07-17

**Authors:** Jianxiong Xu, Zhiming Lv, Zenan Xu, Han Zhang, Mingyu Xia, Wenfang Li

**Affiliations:** 1 School of Pharmaceutical Sciences, Institute of Materia Medica, Xinjiang University, Urumqi 830017, China; jianxiongxu@stu.xju.edu.cn (J.X.); zhiminglv@126.com (Z.L.); xuzenan@stu.xju.edu.cn (Z.X.); hanzhang97@stu.xju.edu.cn (H.Z.); 2 College of Life Science and Technology, Xinjiang University, Urumqi 830017, China; 3 Department of Gastrointestinal Surgery, The Fifth Affiliated Hospital of Xinjiang Medical University, Urumqi 830017, China; 4 School of Clinica Pharmacy, Shenyang Pharmaceutical University, Shenyang 117004, China

**Keywords:** *Strophanthus gratus*, ouabain, microRNA, *miR-145-5p*, colorectal cancer, breast cancer

## Abstract

**Background:** Natural products with unique mechanisms remain of great interest because targeted cancer therapies frequently fail due to toxicity or resistance. Cardiac glycosides have demonstrated antitumor activity, but whether their effects involve microRNA regulation remains largely unexplored. This study investigates whether ouabain derived from *Strophanthus gratus* (Wall. & Hook. ex Benth.) Baill. (SGO) exerts its antitumor effects through *miR-145-5p*, a known tumor suppressor, using both colorectal and breast cancer models. **Methods:** We performed transcriptomic profiling in HCT116 colorectal cancer cells treated with SGO, followed by in vitro assays—including cell viability, caspase 3/7 activity, flow cytometry, and colony formation—in HCT116 and MCF-7 breast cancer cells. In vivo efficacy was evaluated using HCT116 xenograft models in BALB/*c-nu/nu* mice. *miR-145-5p* gain- and loss-of-function approaches were employed to determine its functional requirement. **Results:** SGO dose-dependently suppressed proliferation, induced apoptosis, and inhibited colony formation in both colorectal (HCT116) and breast (MCF-7) cancer cells, and significantly upregulated *miR-145-5p* levels in both cell types. Transcriptomic analysis identified *miR-145-5p* as a highly differentially expressed miRNA. In HCT116 xenograft models, SGO inhibited tumor growth by approximately 60% and elevated intratumoral *miR-145-5p* levels. Importantly, inhibition of *miR-145-5p* significantly attenuated these effects both in vitro and in vivo, establishing that the antitumor activity of SGO depends on the upregulation/activation of *miR-145-5p* in both cancer types. **Conclusions:** We have found that SGO inhibits colorectal and breast cancer growth through a *miR-145-5p*-dependent mechanism, revealing a previously unrecognized regulatory axis for cardiac glycosides. These findings position SGO as a promising candidate for further preclinical studies and suggest that pharmacologic re-expression of *miR-145-5p* may represent a viable therapeutic strategy in targeted therapy.

## 1. Introduction

Cancer remains a significant global health problem, accounting for more than 19 million new cases and 10 million deaths each year, and there is a need for novel treatment strategies that are both effective and safe [1,2]. While some cancers are responsive to traditional cytotoxic chemotherapy, it is not always specific and can lead to systemic toxicity and resistance to treatment [2,3,4]. The field of precision medicine has been revolutionized by targeted therapies, such as monoclonal antibodies and small molecule inhibitors, which have been developed [5,6]; however, these therapies are expensive, have off-target effects and have been associated with the development of drug-resistant mutations [7]. The restrictions highlight the need to investigate alternative therapeutic agents, especially those that are natural products, which have traditionally been a source of important drugs.

Cardiac glycosides are naturally occurring steroidal drugs that are traditionally prescribed to treat heart failure, and have recently been the subject of scientific interest because of their surprising anti-tumor effects [8,9,10]. In particular, preclinical research and retrospective patient data suggest that these compounds have the ability to inhibit the growth of a variety of cancers, including breast, lung, prostate and leukemia [11,12,13]. Of these, ouabain, which was first isolated from the African plant *Strophanthus gratus* (Wall. & Hook. ex Benth.) Baill. is the most important. ouabain has been reported to have strong cytotoxic activity against several cancer cell lines [14]. Ouabain showed potent antiproliferative activity with IC_50_ values of 150 ± 2 nM (24 h) and 90 ± 2 nM (48 h) in MDA-MB-231 breast cancer cells (Winnicka et al., 2007) [15]. Salyer et al. (2013) demonstrated that 1 μM ouabain inhibited the proliferation of various breast cancer cell lines, such as MCF-7, T47D and MDA-MB-231 [16]. The anticancer properties of ouabain have been attributed to various mechanisms such as inhibition of Na^+^/K^+^-ATPase, activation of caspase-3, induction of apoptosis, disturbance of ion homeostasis, and regulation of signaling pathways like Src kinase/phosphoinositide 3-kinase/protein kinase B (Src/PI3K/Akt and Ras-MEK-ERK) pathways [17]. This selective cytotoxicity has been linked to several signaling pathways, such as calcium signaling and Apo-2 ligand/TNF-related apoptosis-inducing ligand (Apo2L/TRAIL)-mediated apoptosis [18]. Busonero et al. (2020) found that ouabain and digoxin stimulate the proteasome and trigger estrogen receptor α(Erα) degradation in breast cancer cells, a novel ‘anti-estrogen’-like mechanism that is independent of the Na^+^/K^+^-ATPase inhibition effect [19]. The repurposing of cardiac glycosides in oncology has been recently reported, with the advantage that they exhibit good safety profiles at anticancer doses and selectively target cancer cells while leaving normal cells unaffected [20]. However, it is still unclear whether cardiac glycosides do so via the regulation of specific MicroRNAs (miRNAs), despite this increasing evidence.

MicroRNAs are small non-coding RNAs that control the expression of genes by binding to the 3′-untranslated regions (UTRs) of target mRNAs to regulate important cellular functions like proliferation, differentiation, and apoptosis [21,22]. It is known that the dysregulation of miRNAs is a characteristic of cancer, and that certain miRNAs act as tumor suppressors or oncogenes, depending on their mRNA targets [13,23]. Of these, *miR-145-5p* has been identified as a critical tumor suppressor that is commonly silenced in colorectal, breast and prostate cancers [24,25]. The restoration of *miR-145-5p* expression suppresses tumor proliferation, invasion and metastasis by targeting oncogenes like *MYC* proto-oncogene (*c-Myc*), Kirsten rat sarcoma viral oncogene homolog (*KRAS*), and fascin actin-bundling protein 1 (*FSCN1*) [26]. In addition to these canonical targets, recent studies show that *miR-145-5p* inhibits breast tumorigenesis by repressing SUMO specific peptidase 2 (SENP2) and thereby inducing SUMOylation-dependent degradation of extracellular signal-regulated kinase 2 (ERK2), which in turn helps to control epithelial–mesenchymal transition (EMT) [27]. Of particular interest, *miR-145-5p* is epigenetically silenced in several cancer types, and re-expression of this miRNA could be clinically useful [28]. Nevertheless, the ability of natural compounds to regulate the activity of *miR-145-5p* and its downstream signaling pathways is still under-explored.

We conducted transcriptome analysis in this study and found that *miR-145-5p* is a crucial player in the anti-tumor mechanism of ouabain from *Strophanthus gratus* (Wall. & Hook. ex Benth.) Baill. (SGO). By applying a multidisciplinary approach that integrates in vitro assays, in vivo xenograft models and genetic manipulation of the *miR-145-5p* expression, we show that SGO inhibits tumorigenesis by *miR-145-5p*-mediated inhibition of oncogenic pathways. These findings reveal a previously unrecognized mechanism for cardiac glycosides and provide a mechanistic rationale for further evaluating SGO in targeted cancer therapy. SGO is a representative natural product with unexplored miRNA-regulating capacity. For cancer models, we focused on colorectal and breast cancers, two of the most prevalent and deadly malignancies worldwide [1], in which the tumor suppressor *miR-145-5p* is frequently downregulated [29]. This makes them suitable for testing the hypothesis that SGO acts through *miR-145-5p*. Specifically, we used the HCT116 colorectal cancer cell line and the MCF-7 breast cancer cell line for several complementary reasons: (i) both cancer types exhibit frequent downregulation of *miR-145-5p*, which provides a common mechanistic basis for evaluating SGO’s miRNA-mediated effects [30,31]; (ii) both are well-characterized, widely used models with stable and reproducible phenotypes that have been extensively employed in studies of miRNA-mediated drug responses [32,33,34,35]; (iii) both cell lines express functional wild-type tumor Protein p53 (p53), which allows direct comparison with previous reports on cardiac glycosides and enables assessment of p53-dependent apoptotic responses [36,37]; (iv) both are driven by different mutations, with HCT116 harboring a *KRAS G13D* mutation characteristic of colorectal cancer and the MCF-7 being estrogen receptor (ER)-positive with intact estrogen signaling, allowing testing of whether SGO’s *miR-145-5p*-dependent mechanism operates across different driver mutations [38,39]; and (v) demonstrating efficacy in both colorectal and breast cancer broadens the potential clinical applicability of our findings, supporting the generalizability of the *miR-145-5p*-mediated antitumor mechanism [40].

## 2. Results

### 2.1. SGO Inhibits Cancer Cell Proliferation and Induces Apoptosis In Vitro

**Figure 1:**
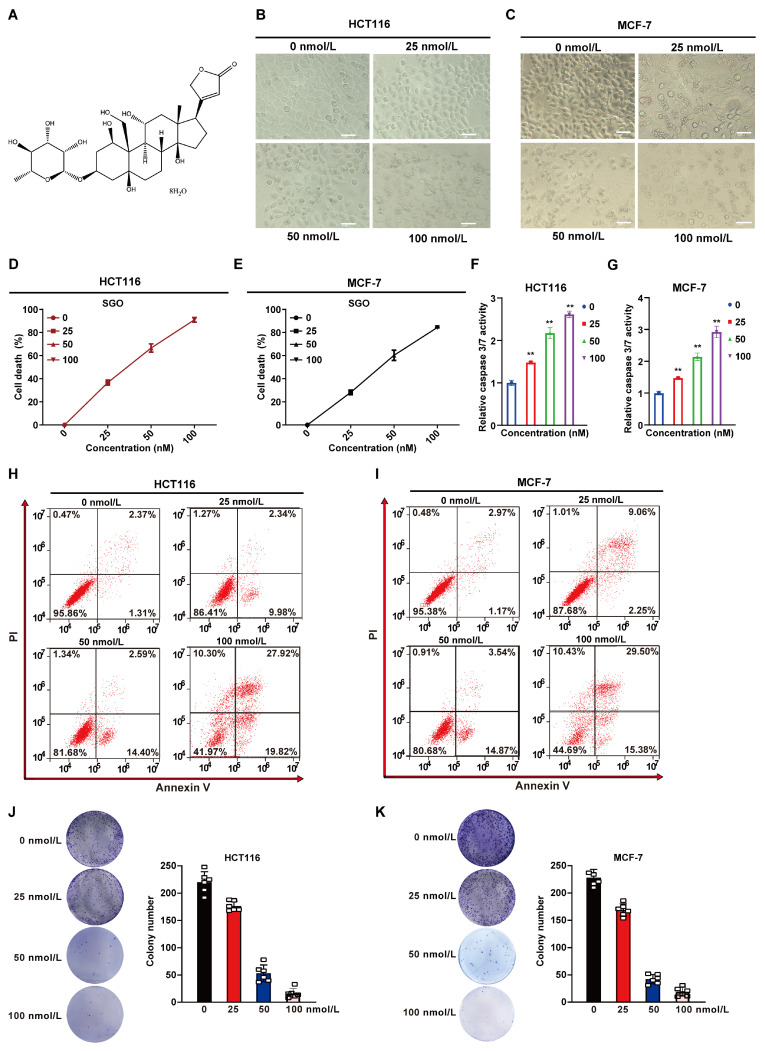
In vitro assays conducted with SGO-treated cancer cells. (**A**) Chemical structure of SGO. Molecular structure of SGO with key functional groups highlighted. (**B**,**C**) Morphological effects of SGO on cancer cells. Micrographs of HCT116 (**B**) and MCF-7 (**C**) cells treated with SGO at concentrations of 0, 25, 50, and 100 nM. Scale bars: 1 μm. Dose-dependent changes in cell density and morphology were observed. (**D**,**E**) Dose-dependent cytotoxicity of SGO. Line graphs depict cell death percentage (*y*-axis) as a function of SGO concentration (*x*-axis) in HCT116 (**D**) and MCF-7 (**E**) cells. (**F**,**G**) Caspase-3/7 activation by SGO. Bar charts illustrate relative caspase-3/7 activity (*y*-axis) in HCT116 (**F**) and MCF-7 (**G**) cells following treatment with SGO (0–100 nmol/L). (**H**,**I**) Apoptosis induction by SGO. Flow cytometry dot plots of HCT116 (**H**) and MCF-7 (**I**) cells. Quadrant percentages represent viable (Q3), early apoptotic (Q4), late apoptotic (Q2), and necrotic (Q1) populations. SGO treatment shifts cell populations toward apoptotic states. (**J**,**K**) Inhibition of colony formation by SGO. Representative images and corresponding bar charts show colony numbers in HCT116 (**J**) and MCF-7 (**K**) cells (*n* = 6). All experiments were performed with *n* = 3 biological replicates. Significance is indicated as follows: ** *p* < 0.01.

The cytotoxicity and apoptosis induced by SGO (Figure 1A) were evaluated in HCT116 and MCF-7 cells that were treated with 0, 25, 50 and 100 nM SGO. Morphological observation (Figure 1B,C), cell viability assays, caspase 3/7 activity measurements, annexin V/propidium iodide (PI) staining by flow cytometry and colony-formation assays were used to assess cell responses. The results demonstrated that cell mortality in HCT116 cells increased in a dose-dependent manner, ranging from approximately 0% at 0 nM to 25% at 25 nM, 60% at 50 nM, and 90% at 100 nM. A similar trend was observed in the MCF-7 cells, with cell death increasing from 0% at 0 nM to 20% at 25 nM, 40% at 50 nM, and 80% at 100 nM (Figure 1D,E). Caspase 3/7 activity in HCT116 cells also increased with increasing SGO concentrations (1-fold at 0 nM, 1.5-fold at 25 nM, 2.5-fold at 50 nM, and 2.8-fold at 100 nM), and the increase was even more pronounced in MCF-7 cells (1-fold at 0 nM, 1.2-fold at 25 nM, 2.2-fold at 50 nM, and 3.5-fold at 100 nM) (Figure 1F,G). Caspase-3/7 are key executioner proteases in the apoptotic cascade; their increased activity directly reflects the induction of apoptosis. Flow cytometry analysis revealed a significant increase in both early (annexin V^+^/PI^−^) and late (annexin V^+^/PI^+^) apoptotic populations following SGO treatment (Figure 1H,I). Furthermore, the colony-formation capacity was markedly reduced with increasing SGO concentration, with colony numbers in both HCT116 and MCF-7 cells dropping to approximately 10 at 100 nM, representing a 95% reduction in comparison with the value at 0 nM (220; Figure 1J,K). These findings indicate that SGO compounds induce significant concentration-dependent cytotoxic and pro-apoptotic effects in HCT116 and MCF-7 cells, potentially mediated through activation of the caspase 3/7 signaling pathway.

### 2.2. Comparative Analysis of DMSO- and SGO-Treated Groups Using Differential Gene Expression Profiling, Gene Ontology and Kyoto Encyclopedia of Genes and Genomes Enrichment Assessments, and miRNA Regulatory Patterns

**Figure 2:**
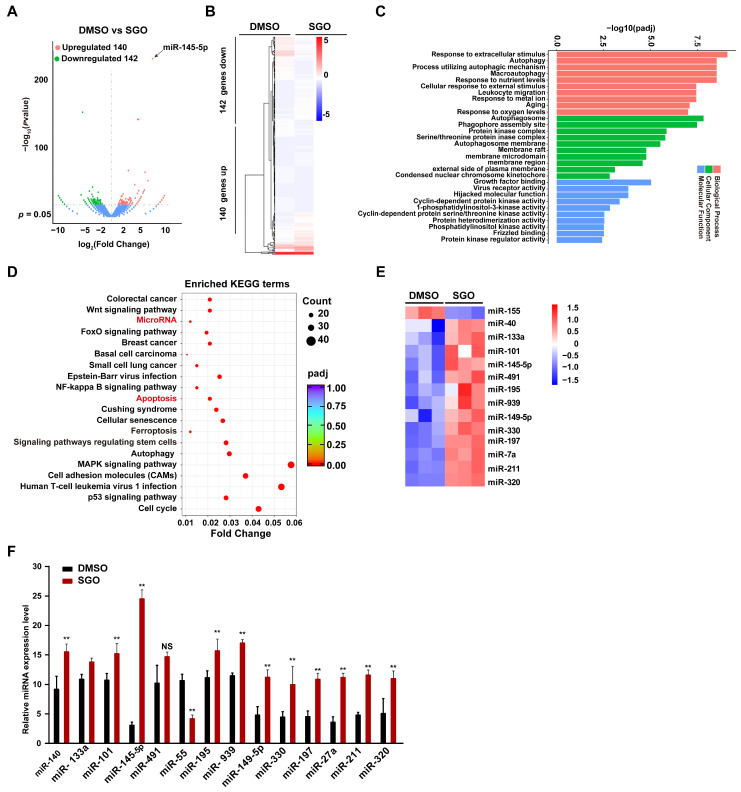
Comparative analysis of DMSO and SGO groups through differential gene expression profiling, GO/KEGG enrichment, and miRNA regulation. (**A**) Volcano plot of differential gene expression. The *x*-axis represents log_2_(fold change), and the *y*-axis indicates −log_10_(*p*-value). Red and green dots represent genes that are significantly upregulated (*n* = 140) and downregulated (*n* = 142), respectively, based on a *p*-value threshold of 0.05. Blue dots represent genes that do not meet the criteria for differential expression (i.e., *p* > 0.05 or below the fold change threshold). Experimental comparison: DMSO vs. SGO. (**B**) Heatmap of gene expression patterns. The color gradient reflects the expression levels across the DMSO and SGO groups. (**C**) Bar chart of enriched biological processes. The *y*-axis lists the biological processes, and the *x*-axis shows −log_10_(padj) values. The bar length corresponds to the level of enrichment significance. Functional categories are color-coded (red/green/blue). (**D**) Scatter plot of KEGG pathway enrichment. The *x*-axis displays the log_2_(fold change), and the *y*-axis lists the enriched KEGG terms. The dot size reflects the number of involved differentially expressed genes, and color represents the padj value. Key cancer-related pathways are labeled. (**E**) Heatmap of miRNA differential expression. The color gradient illustrates miRNA expression changes between the DMSO and SGO groups. (**F**) Bar chart comparing miRNA expression levels. The *x*-axis lists miRNA names, and the *y*-axis shows relative expression levels. Black and red bars represent the DMSO and SGO groups, respectively (*n* = 3). Significance is indicated as follows: ** *p* < 0.01; NS, not significant. Transcriptomic analysis was performed with *n* = 3 biological replicates per group. DEGs were identified using Cuffdiff with FDR ≤ 0.05.

**Figure 3:**
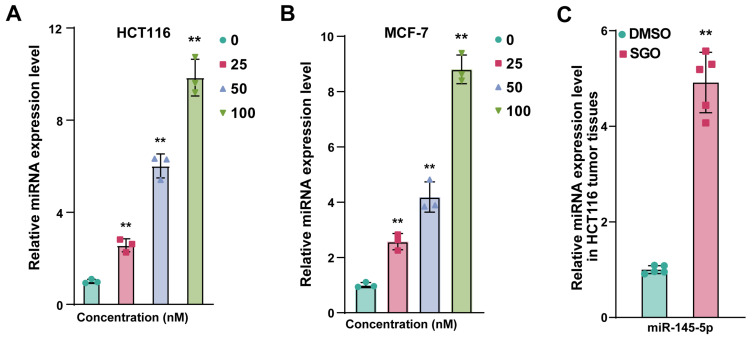
SGO upregulates *miR-145-5p* expression in vitro and in vivo. (**A**,**B**) qRT-PCR analysis of *miR-145-5p* expression in HCT116 (**A**) and MCF-7 (**B**) cells after 48 h of treatment with the indicated concentrations of SGO. Expression levels were normalized to U6 snRNA; values represent fold change relative to the 0 nM control. Data are mean ± SD. (**C**) qRT-PCR analysis of *miR-145-5p* expression in tumor tissues harvested from HCT116 xenograft-bearing mice treated with vehicle control or SGO (10 mg/kg). Each dot represents an individual tumor sample (*n* = 5 per group); Data are mean ± SD from *n* = 3 biological replicates. ** *p* < 0.01 compared with the 0 nM group (Student’s *t*-test).

To investigate the effects of SGO treatment in comparison with DMSO treatment on gene expression, gene expression profiling and miRNA expression analysis were performed by comparing the two groups. The results revealed that, as shown in the scatter plot (Figure 2A), 140 genes were upregulated and 142 genes were downregulated in the SGO group in comparison with the DMSO group, based on a *p*-value threshold of 0.05. Among these, some upregulated genes exhibited log_2_(fold change) values ranging from 5 to 10 and −log_10_(*p*-value) values between 100 and 200, while some downregulated genes showed log_2_(fold change) values from −10 to −5. The heatmap (Figure 2B) illustrates the distinct expression patterns of the upregulated and downregulated genes in the two groups. As shown in the bar chart (Figure 2C), biological processes such as response to extracellular stimulus and autophagy were significantly enriched (high −log_10_(padj) values). The Kyoto Encyclopedia of Genes and Genomes (KEGG) pathways were enriched in the bubble plot (Figure 2D) with bubble size indicating the number of differentially expressed genes and the color indicating the padj value. Interestingly, KEGG pathway enrichment analysis also identified strong links with the mitogen-activated protein kinase (MAPK) and p53 pathways. The enrichment of these cancer-related cascades indicates that SGO could have its anti-tumor activity through the regulation of several oncogenic and tumor-suppressive networks. Specific miRNAs that are differentially expressed between the two groups are shown in a heatmap (Figure 2E). Moreover, the bar chart (Figure 2F) revealed that several miRNAs were significantly up-regulated in the SGO group compared to the DMSO group, particularly *miR-145-5p*. Notably, *miR-145-5p* was selected for further study because of the following: (i) it was the most significantly upregulated miRNA among the cells treated with SGO (fold change > 8, *p* < 0.01); (ii) it is a well-established tumor suppressor that is frequently downregulated in both colorectal and breast cancers [29]; (iii) it targets several oncogenes that are central to cancer pathogenesis, including *c-Myc*, *KRAS*, and *FSCN1* [41,42,43]; and (iv) its upregulation was independently confirmed by Quantitative reverse transcription (qRT)-polymerase chain reaction (PCR) in both HCT116 and MCF-7 cells and in xenograft tumor tissues (Figure 3). Together, these results suggest that SGO treatment significantly affects the gene expression profiles, including modulation of differentially expressed genes (DEGs) involved in biological processes, such as the response to extracellular stimuli and autophagy, as well as pathways related to colorectal cancer, and expression of specific miRNAs, particularly *miR-145-5p*.

### 2.3. SGO Upregulates miR-145-5p In Vitro and In Vivo

To determine whether SGO affects *miR-145-5p* expression, we measured its levels in HCT116 and MCF-7 cells following SGO treatment. qRT-PCR analysis revealed that SGO treatment significantly increased *miR-145-5p* expression in a dose-dependent manner, with a maximum increase of approximately 9.5-fold at 100 nM in both cell lines (Figure 3A,B). Consistent with these in vitro findings, *miR-145-5p* levels were also elevated in tumor tissues from SGO-treated xenograft mice (Figure 3C). These results indicate that SGO effectively upregulates *miR-145-5p* expression in both cultured cancer cells and in vivo.

### 2.4. miR-145-5p Mediates the Antitumor Effects of SGO In Vitro

**Figure 4:**
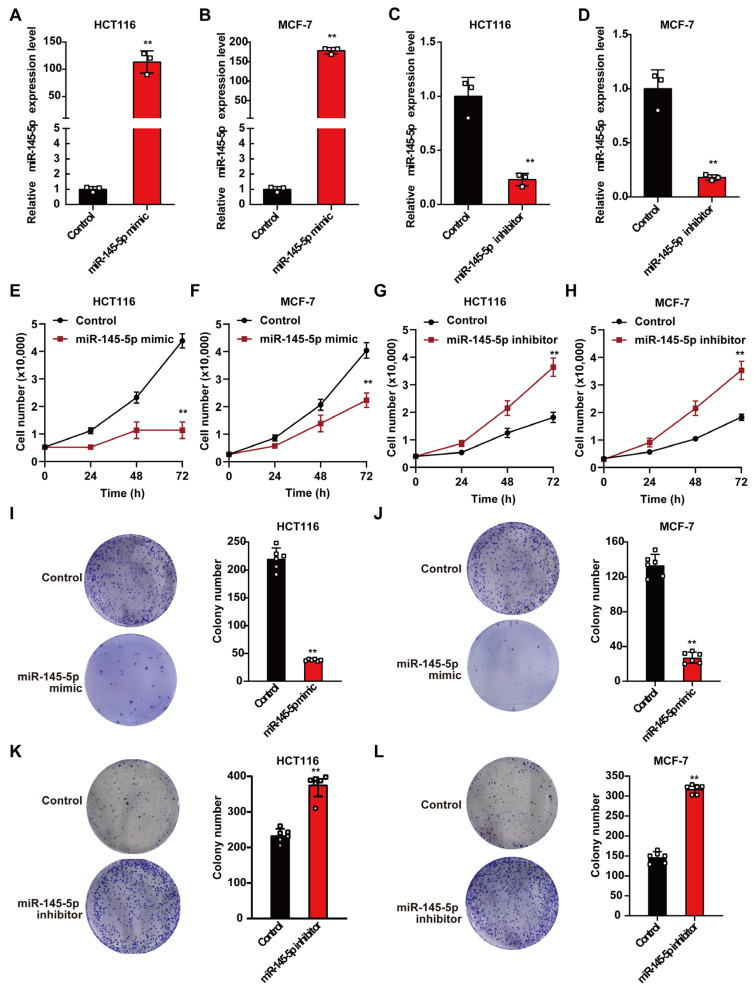
Comparative analysis of the functional effects of *miR-145-5p* using mimic and inhibitor transfection. (**A**,**B**) Validation of *miR-145-5p* overexpression. Bar charts show relative *miR-145-5p* expression levels (*y*-axis) in HCT116 (**A**) and MCF-7 (**B**) cells. (**C**,**D**) Validation of *miR-145-5p* knockdown. Bar charts demonstrate reduced *miR-145-5p* expression in HCT116 (**C**) and MCF-7 (**D**) cells. (**E**,**F**) Viability of HCT116 (**E**) and MCF-7 (**F**) cells following *miR-145-5p* overexpression. (**G**,**H**) Viability of HCT116 (**G**) and MCF-7 (**H**) cells in *miR-145-5p* inhibitor groups. (**I**–**L**) Colony formation assay images and corresponding bar charts quantifying colony numbers in HCT116 (**I**,**K**) and MCF-7 (**J**,**L**) cells (*n* = 6). Significance is indicated as follows: ** *p* < 0.01. Data are mean ± SD from *n* = 3 biological replicates. ** *p* < 0.01 (one-way ANOVA with Tukey’s post hoc test).

To investigate the effects of *miR-145-5p* upregulation or downregulation on the proliferation and colony formation of HCT116 and MCF-7 cells, we treated the cells with an *miR-145-5p* mimic (upregulation) or inhibitor (downregulation), along with a control group. The bar charts (Figure 4A–D) indicated significant upregulation of *miR-145-5p* in the mimic group (approximately 100–150-fold in HCT116 cells, with a similar trend in MCF-7 cells) and significant downregulation in the inhibitor group (approximately 0.1–0.2-fold in HCT116 cells). The line graphs (Figure 4E–H) demonstrated suppressed proliferation in the mimic group (e.g., 2 × 10^4^ cells at 72 h in HCT116 mimic vs. 4 × 10^4^ in control) and enhanced proliferation in the inhibitor group (4.5 × 10^4^), with cell numbers potentially showing visual estimation errors. The bar charts (Figure 4J,L) and colony images (Figure 4I,K) revealed reduced colony numbers in the mimic group (approximately 50 colonies in HCT116 mimic vs. 200 in control) and increased numbers in the inhibitor group (300 colonies), with colony counts potentially showing visual recognition errors. All differences were highly significant (*p* < 0.01). These findings indicated that *miR-145-5p* significantly regulates the proliferation and colony formation of HCT116 and MCF-7 cells, with suppression upon upregulation and promotion upon downregulation.

### 2.5. miR-145-5p Inhibition Attenuates SGO-Induced Antitumor Effects

**Figure 5:**
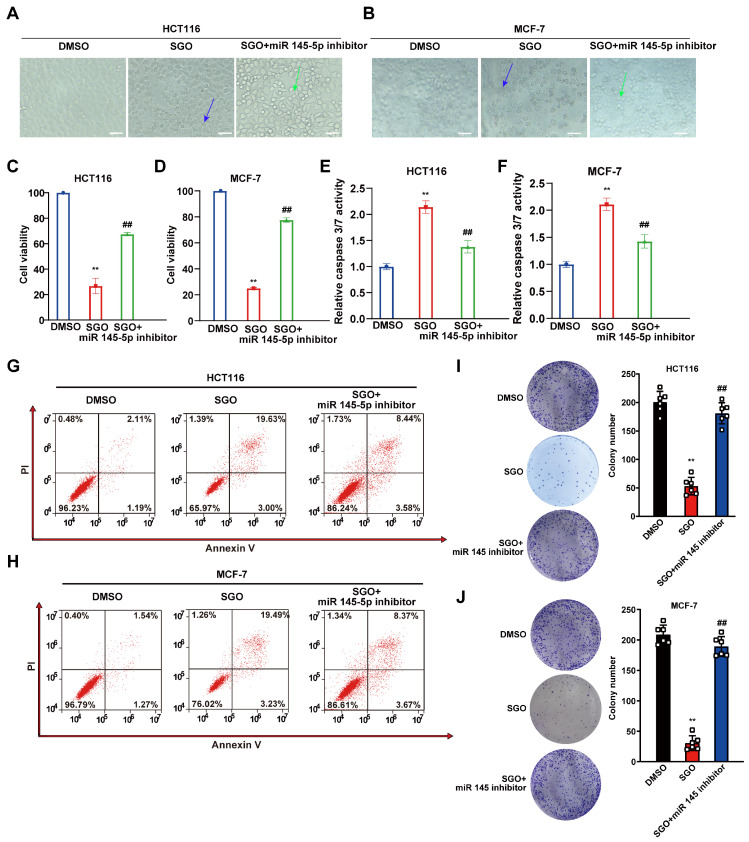
The effects of SGO treatment and *miR-145-5p* inhibition on cancer cells. (**A**,**B**) Cellular morphology under the treatment of SGO. Microscopic images of HCT116 (**A**) and MCF-7 (**B**) cells depicting morphological changes across three groups: DMSO (control, grayscale), SGO (blue arrows indicate apoptotic features), and SGO + *miR-145-5p* inhibitor (green arrows show partial morphological rescue). Scale bars: 1 μm. (**C**,**D**) Cell viability quantification. Bar charts depicting survival rates (%) in HCT116 (**C**) and MCF-7 (**D**) cells. Red bars: DMSO; blue bars: SGO; green bars: SGO + *miR-145-5p* inhibitor. SGO treatment significantly reduces viability (** *p* < 0.01 vs. DMSO), which is partially reversed by *miR-145-5p* inhibition (** *p <* 0.01 vs. SGO). (**E**,**F**) Caspase-3/7 activation dynamics. Bar charts showing relative caspase activity in HCT116 (**E**) and MCF-7 (**F**) cells. SGO treatment (blue bars) significantly induced caspase activation (** *p <* 0.01), which was attenuated by *miR-145-5p* inhibition (green bars, ## *p* < 0.01 vs. SGO). (**G**,**H**) Apoptosis profiling by flow cytometry. Scatter plots of annexin V/PI staining for HCT116 (**G**) and MCF-7 (**H**). Quadrant percentages represent viable (Q3), early apoptotic (Q4), late apoptotic (Q2), and necrotic (Q1) cell populations. SGO treatment increases apoptotic populations (Q2 + Q4), with partial reversal observed upon *miR-145-5p* inhibition. (**I**,**J**) Clonogenic survival assessment. Representative colony images and corresponding bar charts for HCT116 (**I**) and MCF-7 (**J**) cells. SGO treatment (blue bars) significantly decreases colony formation (** *p <* 0.01), which is partially restored by *miR-145-5p* inhibition (green bars, ## *p <* 0.01 vs. SGO) (*n* = 6). Data are mean ± SD from *n* = 3 biological replicates. ** *p* < 0.01 vs. DMSO control; ## *p* < 0.01 vs. SGO alone (one-way ANOVA with Tukey’s post hoc test).

To validate whether the antitumor effects of SGO are mediated through *miR-145-5p*, we treated HCT116 and MCF-7 cells with SGO alone or in combination with an *miR-145-5p* inhibitor. SGO treatment alone significantly reduced cell viability to 78.4 ± 3.1% in HCT116 cells and 76.2 ± 2.8% in MCF-7 cells relative to DMSO control (*p* < 0.01). Co-treatment with the *miR-145-5p* inhibitor partially restored viability to 92.1 ± 2.5% in HCT116 cells and 89.5 ± 3.0% in MCF-7 cells (*p <* 0.01 vs. SGO alone) (Figure 5C,D). As shown in Figure 5A–J, SGO treatment significantly reduced cell viability by approximately 20% in both HCT116 and MCF-7 cells, increased caspase 3/7 activity by approximately 2.0-fold in both cell lines, and decreased colony numbers by approximately 50 in each cell line. Caspase-3/7 activity was increased by 2.3 ± 0.2-fold in HCT116 cells and 2.5 ± 0.3-fold in MCF-7 cells following SGO treatment (*p* < 0.01), whereas this induction was significantly reduced to 1.4 ± 0.1-fold and 1.5 ± 0.2-fold, respectively, upon *miR-145-5p* inhibition (*p* < 0.01 vs. SGO alone) (Figure 5E,F). Flow cytometry analysis revealed that SGO treatment increased the proportion of apoptotic cells (early and late apoptosis combined: 42.3% in HCT116 and 38.7% in MCF-7, vs. 8.2% and 7.5% in DMSO controls), and this effect was partially reversed by co-treatment with the *miR-145-5p* inhibitor (18.5% in HCT116 and 15.2% in MCF-7) (Figure 5G,H). Colony formation assays further confirmed that SGO treatment markedly suppressed clonogenic survival, with colony numbers decreasing from 215 ± 12 in controls to 48 ± 6 in HCT116 cells and from 198 ± 10 to 52 ± 5 in MCF-7 cells (*p* < 0.01). The addition of the *miR-145-5p* inhibitor significantly rescued colony formation (142 ± 10 in HCT116 and 128 ± 9 in MCF-7, *p* < 0.01 vs. SGO alone) (Figure 5I,J). Collectively, these findings demonstrate that SGO exerts antitumor effects through an *miR-145-5p*-dependent mechanism, as inhibition of *miR-145-5p* significantly attenuates SGO-induced suppression of cell viability and colony formation, as well as activation of apoptosis.

### 2.6. SGO Suppresses Tumor Growth in Xenograft Models in an miR-145-5p-Dependent Manner

**Figure 6:**
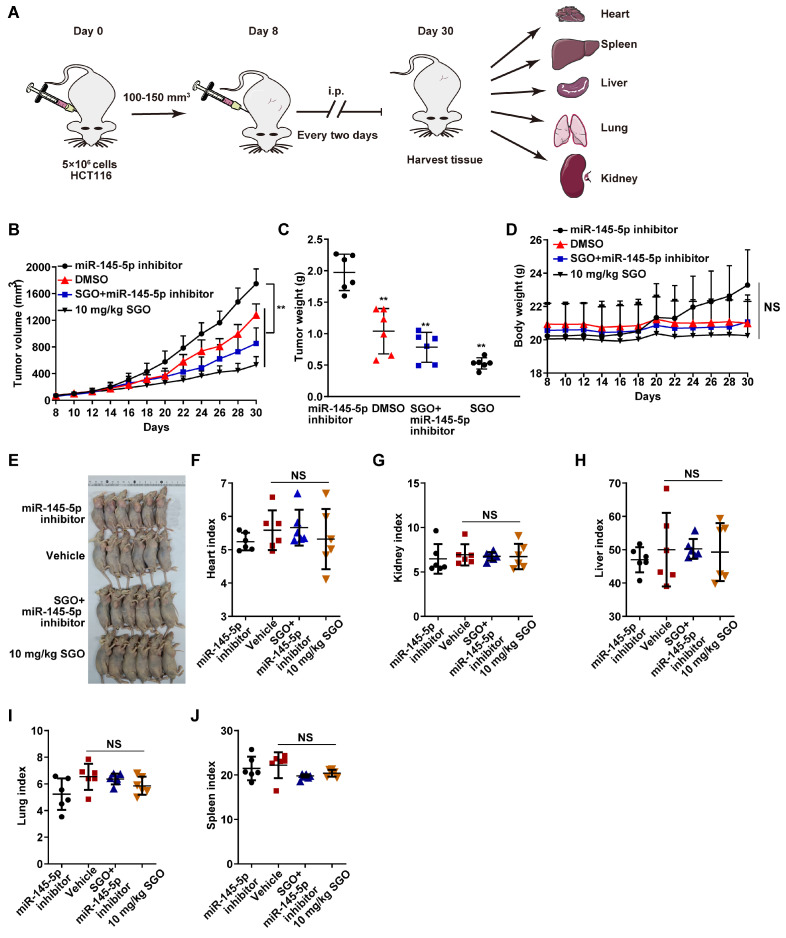
SGO suppresses tumor growth in an *miR-145-5p*-dependent manner in vivo. (**A**) Schematic of the experimental timeline. HCT116 cells were subcutaneously injected into BALB/*c-nu/nu* mice on day 0. Once tumors reached 100–150 mm^3^ (day 8), mice were randomized into four groups (*n* = 6 per group): vehicle control, SGO alone (10 mg/kg, intraperitoneal), SGO combined with intratumoral injection of an *miR-145-5p* inhibitor, and *miR-145-5p* inhibitor alone. Dosing was administered every two days. Mice were euthanized on day 30. (**B**) Tumor growth curves over the treatment period. Data are mean ± SD (*n* = 6 per group). ** *p* < 0.01 compared with control; ** *p* < 0.01 compared with SGO alone. (**C**) Tumor weights at endpoint. Each dot represents an individual tumor; bars indicate mean ± SD. ** *p* < 0.01 vs. control; ** *p* < 0.01 vs. SGO alone. (**D**) Body weight changes throughout the experiment. No significant differences were observed among groups. (**E**) Photographs of mice. (**F**–**J**) Organ indices (heart, liver, spleen, lung, kidney) at endpoint. No significant differences were detected across treatment groups, indicating no overt toxicity at the administered dose (*n* = 6). Data are mean ± SD (*n* = 6 mice per group for in vivo experiments). ** *p* < 0.01 vs. control; (one-way ANOVA with Tukey’s post hoc test), NS indicates not significant (*p* ≥ 0.05).

To evaluate the in vivo antitumor efficacy of SGO and assess the involvement of *miR-145-5p*, we established HCT116 xenograft tumors in BALB/*c-nu/nu* mice (Figure 6A). Mice were treated with vehicle control, SGO (10 mg/kg), or SGO combined with an *miR-145-5p* inhibitor delivered via intratumoral injection. As shown in Figure 6B, SGO treatment significantly suppressed tumor growth, with mean tumor volume reduced by 62.4% compared with the vehicle control group at day 30 (*p* < 0.01). Co-administration of the *miR-145-5p* inhibitor significantly rescued this effect, with tumor volumes 24.8% larger than those in the SGO-alone group (*p* < 0.01) (Figure 6B). Tumor weights followed a similar trend (Figure 6C) Importantly, no significant differences in body weight were observed among treatment groups throughout the experimental period, indicating that SGO treatment did not cause overt systemic toxicity (Figure 6D). Organ index analysis further revealed no significant alterations in heart, liver, spleen, lung, or kidney weights across groups (Figure 6F–J); however, it should be noted that these macroscopic evaluations alone are insufficient to rule out specific functional toxicities, such as cardiotoxicity.

### 2.7. Clinical Relevance of miR-145-5p Expression in Cancer Patients

To contextualize our findings, we examined *miR-145-5p* expression in publicly available cancer datasets. Consistent with previous reports, The Cancer Genome Atlas (TCGA) analysis revealed no significant correlation between endogenous *miR-145-5p* expression levels and overall survival in colorectal cancer patients (Supplementary Figure S1), suggesting that its tumor-suppressive function may be context-dependent or regulated at the post-transcriptional level.

## 3. Discussion

In this study, we found *miR-145-5p* as a key mediator of the anticancer activity of SGO. We show that the antitumor activity of SGO is mediated by a novel mechanism of transcriptional induction of the *miR-145-5p*, which is supported by transcriptomic profiling, in vitro functional assays, and in vivo xenograft models. This conclusion is confirmed by the observation that the inhibition of *miR-145-5p* dramatically reduces the suppression of cell proliferation, colony formation and tumor growth induced by SGO, while also inhibiting the activation of apoptosis induced by SGO.

Our results complement the previous studies on cardiac glycosides in several ways. To begin with, although these compounds have been shown to be associated with apoptosis and pathway modulation, our transcriptomic analysis offers a more detailed view by pinpointing *miR-145-5p* as a specific upstream regulator. These pathways are also enriched in our KEGG analysis (Figure 2D), and known functions of *miR-145-5p* include targeting of key components of these pathways [44]. These observations indicate candidate mechanisms that can be directly tested in future studies. In addition to Wingless/Integrated (Wnt) signaling, we found significant enrichment of MAPK and p53 pathways by KEGG analysis (Figure 2D). *miR-145-5p* negatively regulates cell proliferation and survival by targeting the *KRAS* and other effectors of the MAPK signaling pathway. In addition, the p53 pathway is a key tumor suppressor pathway that may be indirectly regulated by *miR-145-5p*, and is responsible for the apoptotic effects seen after SGO treatment. These enrichments further support the conclusion that SGO coordinates a broad antitumor transcriptional program via *miR-145-5p*. Second, our in vivo data show that the anti-tumor effect of SGO is reproducible in a physiologically relevant environment, and is critically determined by *miR-145-5p* expression, as shown by the rescue observed when *miR-145-5p* is inhibited. The downstream pathways involved in the antitumoral activity of *miR-145-5p* need to be discussed. *miR-145-5p* is a well-characterized tumor suppressor that directly targets multiple oncogenes. The suppression of *c-Myc* by *miR-145-5p* supports the decreased proliferation and colony formation seen following SGO treatment, and is consistent with computational and experimental studies of *miR-145-5p* binding to the 3′-UTR of *c-Myc* mRNA to suppress its translation and attenuate cell cycle progression and proliferation [41,45]. *c-Myc* is a master regulator that is frequently overexpressed in colorectal and breast cancer. Also, *miR-145-5p* directly targets *KRAS*, an important oncogene in the MAPK signaling pathway. The *KRAS G13D* mutation is present in HCT116 cells and suppression of *KRAS* expression by *miR-145-5p* would be predicted to reduce downstream Raf-mitogen-activated protein kinase kinase-extracellular signal-regulated kinase (Raf-MEK-ERK) signaling, leading to the observed antiproliferative and pro-apoptotic effects [46]. This is consistent with our KEGG enrichment analysis, which revealed that the MAPK signaling pathway was significantly modulated in response to SGO treatment (Figure 2D). *miR-145-5p* also regulates fascin-1 (*FSCN1*), an actin-bundling protein essential for cancer cell migration and invasion, whose downregulation by *miR-145-5p* helps to inhibit the metastatic potential [43]. In addition, *miR-145-5p* inhibits Wnt/beta-catenin signaling by targeting transcription factor 4 (TCF4) and low-density lipoprotein receptor-related protein 6 (LRP6), which are essential factors in this oncogenic pathway that is often dysregulated in colorectal cancer [47]. The p53 pathway could also be indirectly modulated, since both HCT116 and MCF-7 cells have wild-type p53, and *miR-145-5p* could regulate the p53-mouse double minute 2 (MDM2) feedback loop and thus the activation of caspase 3/7 in our study and induction of apoptosis [48]. Our transcriptomic analysis and literature support these proposed mechanisms, but we recognize that these were not directly tested in this study (luciferase reporter assays and Western blot analyses of target protein levels). Future studies are needed to directly test these mechanisms.

Our findings have clinical relevance. Endogenous *miR-145-5p* expression is not associated with overall survival in publicly available datasets (Supplementary Figure S1), but this does not reduce the therapeutic potential of pharmacologically reactivating this tumor suppressor. Our data show that SGO can indeed increase *miR-145-5p* to a level that is enough to inhibit tumor growth, thus pharmacologically targeting *miR-145-5p* is a possible solution to addressing the endogenous expression limitations. The idea of “epigenetic reactivation” appears to be a promising approach for cancer treatment, especially in cases of tumors where *miR-145-5p* is silenced, but still fully functional.

There are some limitations to this study that should be noted. The upstream signals that connect SGO to the *miR-145-5p* transcriptional activation have not yet been clarified. We have shown that *miR-145-5p* is required to mediate the antitumor activity of SGO, but the upstream signaling pathway that connects SGO treatment with *miR-145-5p* transcriptional upregulation is still not well understood and is an important avenue for future research. To further define the therapeutic context of SGO, we re-examined the reported differential sensitivities to ouabain across various cellular models. While a degree of selectivity is consistently observed, the divergent cytotoxicity profiles are largely attributable to cellular heterogeneity and experimental context. Clifford and Kaplan (2013) demonstrated that MCF-7 and MDA-MB-231 cells exhibit marked resistance under standard 72-hr viability assays, contrasting sharply with the sensitivity of non-tumorigenic MCF-10A cells (Table 1) [49]. This apparent discrepancy is partly reconciled by Gould et al. (2018), who documented rapid oncotic lysis of malignant cells within seconds under electroporation-enhanced conditions—a paradigm distinct from passive drug exposure [50]. The existence of a concentration threshold (~1 µM) for broad cytotoxicity, as defined by Salyer et al. (2013), further clarifies these variances. Notably, MDA-MB-453 represents a mechanistic outlier, relying exclusively on V-ATPase for ion homeostasis due to a complete loss of Na,K-ATPase expression [16]. Beyond ion transport, mechanistic divergence extends to downstream effectors: ouabain triggers proteasome-mediated ERα degradation in breast cancer cells [19], whereas SGO selectively targets Nucleoside diphosphate linked moiety X hydrolase 21 (NUDT21) to suppress YAP1 in colorectal models without perturbing normal colon organoids [51]. Collectively, these data suggest that SGO-induced cytotoxicity is not binary, but rather emerges from the convergence of drug concentration, cellular bioenergetics—specifically the complement of ion pumps—and the expression of specific oncogenic drivers such as NUDT21 and ERα. Together, these findings demonstrate that cardiac glycosides including SGO possess a therapeutic window that allows selective targeting of cancer cells at nanomolar concentrations well below those that significantly affect non-malignant cells. In addition, one of the most important factors to be taken into account for the clinical translation of cardiac glycosides is their potential cardiotoxicity and selectivity towards tumor cells. SGO has been reported to have average well-tolerated plasma concentrations in vivo comparable to or even higher than the nanomolar IC_50_ value necessary to effectively inhibit cancer cell growth, although it has potential cardiotoxic properties with chronic or high dose administration (such as early electromechanical remodeling [52] or activation of NOD-like receptor family pyrin domain containing 3 (NLRP3) inflammasome under inflammatory conditions [53]). its average well-tolerated plasma concentration in vivo has been reported to be comparable to or even higher than the nanomolar IC_50_ value required to effectively inhibit cancer cell growth [54,55]. Therefore, the safety of SGO is very relative. The use of it should be carefully limited to non-inflammatory situations, and treatment duration and cumulative doses should be carefully monitored to prevent cardiotoxicity. As long as these strict conditions are kept, SGO has a therapeutic window that enables selective induction of apoptosis in specific cancer cells at sub-clinical doses without harming normal physiological cells [56]. For example, recent studies have shown that SGO has a significant effect on the survival of HCT116 cells, but not on the survival of CRL-1790 normal colon epithelial cells or colorectal organoids [51] and even has cytoprotective effects on human umbilical vein endothelial cells (HUVECs) [57]. This therapeutic window made us want to further explore its anti-proliferative activity against colorectal and breast cancers. The concentrations of SGO that inhibit the proliferation of HCT116 and MCF-7 cells in our study (IC_50_ = 17.2 nM and 19.0 nM, respectively) are around 50–100 fold lower than the micromolar concentrations that are associated with significant cardiotoxicity. Botelho et al. (2020) reported that ouabain has concentration-dependent cardiac effects and that the extent of cardiotoxicity was mainly at micromolar concentrations [52]. Likewise, Kobayashi et al. (2017) showed that ouabain is able to trigger the NLRP3 inflammasome and induce cardiac inflammation at concentrations above 1 uM [53]. Calderon-Montano et al. (2014) highlighted the need to find a therapeutic window to maximize the anticancer effects while minimizing cardiotoxicity during the clinical development of cardiac glycosides [55]. Further structural modification of ouabain to SGO (glycosylation at the 3-position) could lead to an even greater therapeutic index, due to the changes in the pharmacokinetic properties and tissue distribution [58,59]. In line with this safety profile, no significant changes in heart weight, organ indices or body weight were observed in our in vivo studies at an effective dose of 10 mg/kg, indicating no overt acute systemic toxicity. However, the selectivity of cardiac glycosides may also be very tissue-specific, with non-tumorigenic breast cells (MCF-10A) being more sensitive to SGO than some breast tumor cells [49,60,61,62]. Thus, further preclinical studies with extensive cardiac safety assessment (including electrocardiography, arrhythmia analysis, serum troponin levels, etc.) and parallel studies with non-malignant cell lines (MCF-10A, breast cancer; CRL-1790, colorectal cancer) would be beneficial to comprehensively define the therapeutic index of SGO. It should also be emphasized that no overt acute toxicity was observed in vivo at 10 mg/kg, but these macroscopic evaluations (body weight, organ indices) are not sufficient to completely rule out cardiotoxicity and more sensitive functional evaluations will be needed prior to clinical translation.

## 4. Materials and Methods

### 4.1. Cell Lines and Cell Culture

Human colorectal cancer cell line HCT116 and breast cancer cell line MCF-7 were purchased from the Cell Bank of the Chinese Academy of Sciences (Shanghai, China). Both cell lines were cultured in Dulbecco’s modified Eagle’s medium (DMEM, Gibco, Thermo Fisher Scientific, Waltham, MA, USA) supplemented with 10% fetal bovine serum (FBS, Biological Industries, Beit Haemek, Israel) and 1% penicillin–streptomycin (Penicillin-Streptomycin, Gibco, Thermo Fisher Scientific, Waltham, MA, USA). All cells were maintained in a humidified incubator at 37 °C with 5% CO_2_. Routine testing using a Mycoplasma Detection Kit-QuickTest (Biotool, Houston, TX, USA) confirmed that all cell lines were negative for mycoplasma contamination.

### 4.2. SGO Preparation and Stock Solution

SGO (ouabain octahydrate) was purchased from MedChemExpress (MCE, Monmouth Junction, NJ, USA; Cat. No. HY-B0542; CAS No. 11018-89-6). The compound is a white crystalline solid with a molecular weight of 728.77 g/mol (C_29_H_44_O_12_·8H_2_O) and is known to be a selective Na^+^/K^+^-ATPase inhibitor. The manufacturer confirmed its purity (≥98% by HPLC) and identity (by MS and NMR). For in vitro use, a 10 mM stock solution was prepared by dissolving the compound in DMSO. For in vivo administration, the stock solution was further diluted in vehicle consisting of DMSO:PEG300:Tween-80:saline (10:40:5:45, *v*/*v*/*v*/*v*) to achieve the desired final concentration.

### 4.3. miR-145 Inhibition by Transfection

The miR-145 inhibitor was purchased from GenePharma Co., Ltd. (Shanghai, China). The miR-145 inhibitor and Lipofectamine 2000 were separately diluted in serum-free Dulbecco’s modified DMEM medium and incubated at room temperature for several minutes. The two solutions were gently mixed and allowed to stand for 20 min before addition to the cells. Cells transfected with the miR-145 inhibitor were further incubated for 48 h at 37 °C in a CO_2_ incubator before being used in subsequent experiments.

### 4.4. Cell-Viability Assay

For SGO treatment, cells were seeded into 96-well plates at a density of 5 × 10^3^ cells/well. After overnight adhesion, cells were treated with 0, 25, 50, or 100 nM SGO (unless otherwise specified) or vehicle control for 48 h. Cell proliferation was evaluated using the MTS assay as follows: (4,5-dimethylthiazol-2-yl)-5-(3-carboxymethoxyphenyl)-2-(4-sulfophenyl)-2H-tetrazolium (MTS; Promega Corporation, Madison, WI, USA) according to the manufacturer’s instructions. The absorbance was measured at 490 nm using a microplate reader (BioTek Synergy H1; Cat. No. SYNERGYH1; BioTek Instruments, Winooski, VT, USA), and half-maximal inhibitory concentration (IC_50_) values were determined using GraphPad Prism 9.0 (GraphPad Software, San Diego, CA, USA) [63].

### 4.5. Apoptosis Analysis

HCT116 and MCF-7 cells were seeded in 6-well plates at a density of 3 × 10^5^ cells/well. After 24 h, cells were treated with SGO (0, 25, 50, or 100 nM) for an additional 48 h. Apoptosis was then assessed using annexin V/PI staining. Cells were harvested with trypsin (without EDTA), washed with PBS, and resuspended in 1× Binding Buffer (2–5 × 10^5^ cells/mL). For each sample, 195 µL of cell suspension was mixed with 5 µL Annexin V-FITC (NeoBiosciences, Shanghai, China) and incubated at room temperature for 10 min in the dark. Then, 190 µL Binding Buffer and 10 µL PI (20 µg/mL) were added. Stained cells were analyzed immediately on a BD FACSCanto II flow cytometer (excitation/emission: 488/530 nm for FITC, 488/585 nm for PI). At least 10,000 events per sample were recorded. Quadrant gates were set using single-stained and unstained controls. Early apoptotic cells: Annexin V^+^/PI^−^; late apoptotic: Annexin V^+^/PI^+^; necrotic: Annexin V^−^/PI^+^. Experiments were performed in triplicate. Data were analyzed with FlowJo 10. 8. 1.

### 4.6. Colony Formation Assay

Cells were treated with SGO or vehicle control, as described above. Cells were seeded into six-well plates at a density of 500 cells/well and cultured for 8 days. The colonies were fixed with 4% paraformaldehyde and stained with methylene blue. Colony formation was quantified by counting the number of colonies formed. All colony counts were performed by an investigator blinded to the experimental conditions.

### 4.7. Caspase-3/7 Activity

HCT116^WT^ cells were seeded into 96-well white-walled plates at a density of 1 × 10^4^ cells/well and then treated with SGO or vehicle control, as described above. After treatment, the plates were equilibrated to room temperature for 10 min. The Caspase-Glo^®^ 3/7 Reagent was prepared fresh by transferring the entire contents of the Caspase-Glo^®^ 3/7 Buffer bottle into the amber bottle containing the lyophilized Caspase-Glo^®^ 3/7 Substrate (Promega Corporation, Madison, WI, USA, Cat. No. G8091), followed by swirling until the substrate was completely dissolved. Then, 100 μL of the reconstituted reagent was added to each well (1:1 ratio of reagent to sample volume). The plate was gently mixed on an orbital shaker at 300–500 rpm for 30 s and then incubated at room temperature for 1 h. Luminescence was measured using a GloMax^®^ Multi+ Microplate Luminometer (Promega Corporation, Madison, WI, USA) with an integration time of 0.5–1 s per well. A blank control (culture medium without cells) and a negative control (vehicle-treated cells) were included in each plate. caspase-3/7 activity was expressed as relative luminescence units (RLU) after subtracting the blank value. All measurements were performed in triplicate.

### 4.8. RNA Sequencing and Transcriptomic Analysis

HCT116^WT^ cells were treated with SGO or vehicle control as described above. Total RNA was extracted and subjected to RNA sequencing by the Novogene Technology Corporation (Beijing, China) using an Illumina HiSeq 2500 platform (Illumina, San Diego, CA, USA), with three biological replicates per group. Raw reads were pre-processed by removing rRNA sequences, adapter contaminants, short fragments, and low-quality reads. Tophat v2.1.0 was used to align the cleaned reads to the human reference genome (GRCh38/hg38), allowing up to two mismatches. After alignment, Cufflinks v2.1.1 was employed with reference annotations to calculate fragments per kilobase of transcript per million mapped reads (FPKM) values for known gene models. DEGs were identified using Cuffdiff software, version 2.2.1. The statistical significance threshold for DEGs was determined based on a false discovery rate (FDR) ≤ 0.05. Fold changes were calculated on the basis of the FPKM values for each sample [64].

### 4.9. RNA Extraction and Quantitative Reverse Transcription Polymerase Chain Reaction

Total RNA was extracted using the TRIzol reagent (Invitrogen, Thermo Fisher Scientific, Carlsbad, CA, USA) according to the manufacturer’s instructions. One microgram of total RNA was reverse-transcribed into complementary DNA (cDNA) using the PrimeScript Reagent Kit with gDNA Eraser (Takara Bio Inc., Shiga, Japan). qRT-PCR was performed using the SYBR Premix Ex Taq (Takara Bio, Shiga, Japan) in a quantitative PCR (qPCR) system. qPCR was performed using SYBR Fast qPCR Mix for hsa-*miR-145-5p* [65]. The primer sequences were as follows: hsa-*miR-145-5p*: F–GTCCAGTTTTCCCAGGAATCCCT, R–TGGTGTCGTGGAGTCG; U6 snRNA: F–CTCGCTTCGGCAGCACA, R–AACGCTTCACGAATTTGCGT (used as an internal control). For *miR-145-5p* and U6 snRNA detection, stem-loop reverse transcription was performed using specific stem-loop primers. Samples were amplified under the following cycling conditions: 95 °C for 5 min, 95 °C for 30 s, followed by 35 cycles of 60 °C for 30 s and 72 °C for 30 s. After PCR amplification, relative expression levels of *miR-145-5p* were quantified using the 2^−ΔΔCq^ method.

### 4.10. Animals and Ethics Statement

BALB/*c-nu/nu* mice (male; body weight, 18–22 g; age, 6 weeks) were purchased from the Animal Laboratory Center of Xinjiang University (Urumqi, Xinjiang, China) and housed in a temperature-controlled light-cycled animal facility at Xinjiang University. All animal experiments were approved by the Ethics Committee for Animal Experiments of the Xinjiang Key Laboratory of Biological Resources and Genetic Engineering (permit no.: BRGE-AE001), and conducted in accordance with the guidelines of the Animal Care and Use Committee of the College of Life Science and Technology, Xinjiang University. All efforts were made to minimize animal suffering.

### 4.11. Animal Experiments

For the xenograft experiments, BALB/*c-nu/nu* mice were subcutaneously injected with 5 × 10^6^ HCT116^WT^ cells. The tumors were allowed to grow for 8 d until the volume reached 100–150 mm^3^. Mice were then randomly divided into four groups (*n* = 6 per group): vehicle control, SGO alone (10 mg/kg, intraperitoneal), SGO combined with intratumoral injection of an *miR-145-5p* inhibitor, and *miR-145-5p* inhibitor alone. The vehicle solution consisted of a mixture of DMSO:Tween-80:propanediol:phosphate-buffered saline (1:1:1:7, *v*/*v*/*v*/*v*). Tumor size (V) was measured using calipers every 2 days before treatment and daily after treatment initiation, and calculated using the formula V = a × b^2^/2, where a and b represent the major and minor tumor diameters, respectively. The mice were euthanized 30 days after tumor cell inoculation (i.e., 22 days after treatment initiation) [66]. The investigator was blinded to the allocation of the experimental groups during the assessment.

### 4.12. Statistical Analysis

All quantification results are presented as mean ± standard deviation (SD; *n* = 3; unless otherwise specified). For xenograft experiments, statistical analysis was conducted using one-way analysis of variance (ANOVA). For comparisons involving two groups, statistical significance was determined using Student’s *t*-test. For experiments with three or more groups, one-way ANOVA followed by Tukey’s post hoc test was used for multiple comparisons. All data are presented as mean ± SD unless otherwise specified. A *p*-value < 0.05 was considered statistically significant. Statistical analyses were performed using GraphPad Prism 9.0 (GraphPad Software, San Diego, CA, USA).

## 5. Conclusions

**Figure 7:**
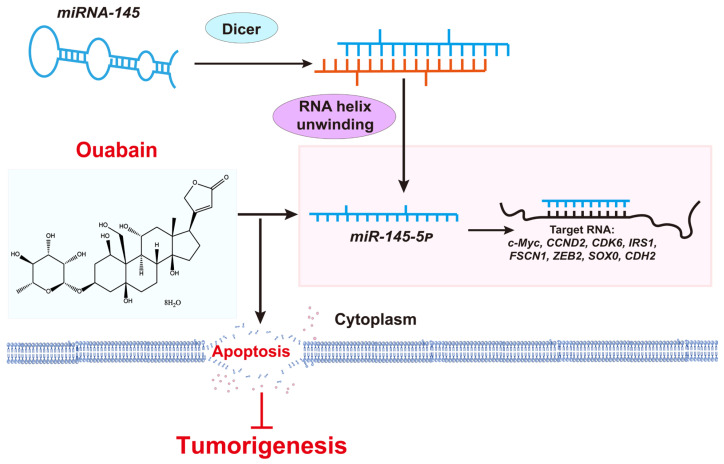
This schematic diagram illustrates the proposed molecular mechanism through which SGO regulates tumor progression via *miR-145-5p*. SGO, a bioactive compound, induces the expression of *miR-145-5p*. Subsequently, the mature miRNA is transported into the cytoplasm, where it modulates key apoptotic signaling pathways. Enhanced apoptosis mediated by *miR-145-5p* contributes to the suppression of tumorigenesis, Black sharp arrows indicate promotion or activation, whereas red flat arrows indicate inhibition.

In conclusion, this study identifies the *miR-145-5p* pathway as a key mechanistic axis through which SGO suppresses tumorigenesis. Using gain- and loss-of-function approaches, we demonstrate that the antitumor efficacy of SGO is dependent on the activation of this tumor-suppressive miRNA. These findings provide a mechanistic rationale for repurposing SGO in oncology and identify a pharmacological inducer of *miR-145-5p* with potential therapeutic relevance (Figure 7). Future studies aimed at elucidating the upstream signaling events linking SGO to *miR-145-5p* activation, as well as comprehensive toxicological evaluation, will be important for translating these findings toward clinical application.

## Figures and Tables

**Table 1:** IC_50_ Values of Ouabain in Breast Cell Lines Data source: Clifford and Kaplan, 2013, PLoS One [49].

Cell Line	Cell Type	Malignancy	IC_50_ (nM)	SE
184D	HMEC	Non-tumorigenic	1354.18	155.1
184A1	HMEC	Non-tumorigenic	1494.1	45.3
MCF-10A	HMEC	Non-malignant	2170.47	192.30
MCF-7	BCC	Low malignant	NE	-
MDA-MB-231	BCC	High malignant	NE	-
MCF10CA1	BCC	Non-malignant	2667.1	574.1

Notes: IC_50_ values represent the concentration (nM) causing 50% reduction in cell viability after 48 h treatment; SE: standard error; NE: not estimable (IC_50_ could not be calculated within the tested concentration range). HMEC: human mammary epithelial cell; BCC: breast cancer cell.

## Data Availability

The supporting the findings of this study is available upon reasonable request from the corresponding author.

## Supplementary Materials

The following supporting information can be downloaded at: https://www.mdpi.com/article/10.3390/ph19071099/s1, Figure S1: Clinical relevance of *miR-145-5p* expression in cancer patients.
