# Supplementary material for: miR-145-5p Is Required for the Antitumor Activity of Strophanthus gratus-Derived Ouabain in Colorectal and Breast Cancer
Source: Pharmaceuticals (Basel). 2026 Jul 17;19(7):1099. doi: 10.3390/ph19071099 (PMC13415168; doi:10.3390/ph19071099)
Supplement: Supplementary file 1 [file pharmaceuticals-19-01099-s001.zip › pharmaceuticals-4368005-supplementary.pdf]

Supporting information

miR-145-5p is required for the antitumor activity of _Strophanthus gratus_-derived ouabain in colorectal and breast cancer

Jianxiong Xu [1,2,†], ZhiMing Lv [1,3,†], ZeNan Xu [1,2], Han Zhang [1,2], MingYu Xia [4,*], WenFang Li [1,2,3,*]

[1] School of Pharmaceutical Sciences and Institute of Materia Medica, Xinjiang University, Urumqi 830017, China;

[2] College of Life Science and Technology, Xinjiang University, Urumqi 830017, China;

[3] Department of Gastrointestinal Surgery, The Fifth Affiliated Hospital of Xinjiang Medical University, Urumqi 830017, China;

[4] School of Clinical Pharmacy, Shenyang Pharmaceutical University, Shenyang 117004, China.

*Correspondence: liwenfang@xju.edu.cn (W.L.); xmywd@vip.sina.com (M.X.)

†These authors contributed equally to this work

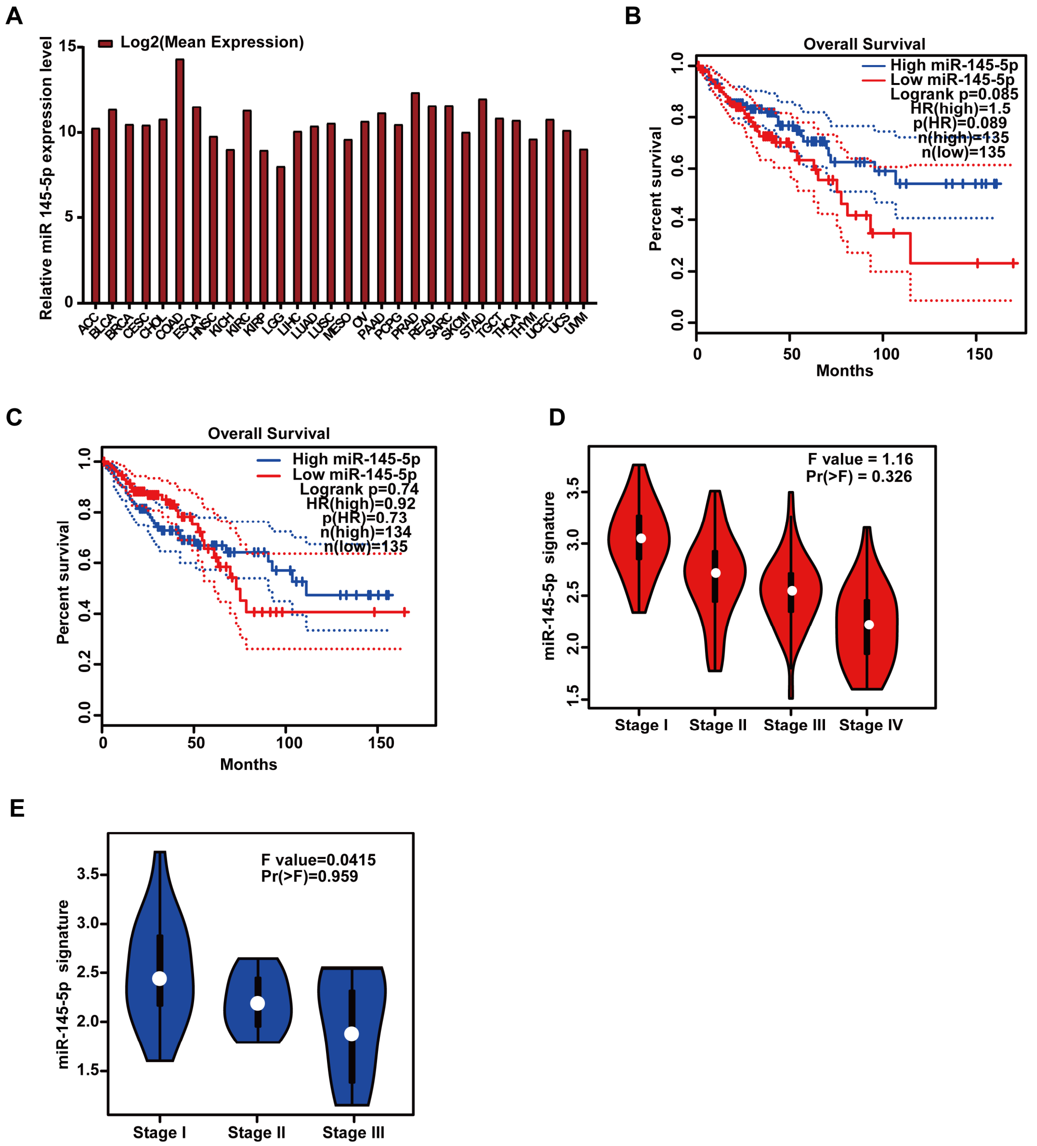

Supplementary Figure S1. Clinical relevance of miR-145-5p expression in cancer patients. (A) miR-145-5p expression profile across TCGA cancer samples. (B–C) Kaplan-Meier analysis of overall survival in patients with high versus low miR-145-5p expression. (D–E) Distribution of miR-145-5p expression across cancer stages. Data were derived from TCGA datasets.
